# Short tail stories: the hirudin-like factors HLF6 and HLF7 of the Asian medicinal leech, *Hirudinaria manillensis*

**DOI:** 10.1007/s00436-021-07316-3

**Published:** 2021-10-02

**Authors:** Christian Müller, Chantal Eickelmann, Dana Sponholz, Jan-Peter Hildebrandt

**Affiliations:** Animal Physiology and Biochemistry, Zoological Institute and Museum, University of Greifswald, Felix-Hausdorff-Str. 1, 17489 Greifswald, Germany

**Keywords:** Hirudin, Hirudin-like factors, Blood coagulation, Medicinal leeches

## Abstract

The leech-derived hirudins and hirudin-like factors (HLFs) share a common molecule structure: a short N-terminus, a central globular domain, and an elongated C-terminal tail. All parts are important for function. HLF6 and HLF7 were identified in the Asian medicinal leech, *Hirudinaria manillensis*. The genes of both factors encode putative splice variants that differ in length and composition of their respective C-terminal tails. In either case, the tails are considerably shorter compared to hirudins. Here we describe the functional analyses of the natural splice variants and of synthetic variants that comprise an altered N-terminus and/or a modified central globular domain. All natural splice variants of HLF6 and HLF7 display no detectable thrombin-inhibitory potency. In contrast, some synthetic variants effectively inhibit thrombin, even with tails as short as six amino acid residues in length. Our data indicate that size and composition of the C-terminal tail of hirudins and HLFs can vary in a great extent, yet the full protein may still retain the ability to inhibit thrombin.

## Introduction

The hirudin-like factors (HLFs) represent an only recently described subclass of compounds derived from the salivary glands of medicinal leeches (Müller et al. 2016, 2017). HLFs comprise structural (e.g., six cysteine residues within a central globular domain) and genetic (a gene structure composed of four exons and three introns) features that are characteristic for hirudins but may considerably differ from hirudins and among each other in biochemical properties like molecular weight (MW) or isoelectric point (*pI* value). In previous works, we have described and functionally characterized several HLFs including HLF1-4 (originating from representatives of the three European medicinal leeches *Hirudo medicinalis*, *Hirudo verbana*, and *Hirudo orientalis*) (Müller et al. 2016, 2020a, b) and HLF5, HLF6, and HLF8 (originating from *Hirudinaria manillensis*, an Asian medicinal leech) (Lukas et al. 2019), respectively. Whereas HLF2-4 and HLF6 did not exhibit any measurable thrombin-inhibitory potency, some variants of HLF1 and both HLF5 and HLF8 did. In addition, we have constructed and functionally characterized synthetic hybrid variants of all four HLFs (HLF1-4) of *Hirudo* sp. Our data clearly indicated that all three parts of the HLF molecules (the N-terminus, the central globular domain, and the C-terminus) are of crucial importance for their ability to inhibit thrombin. Slight modifications within the N-termini converted HLF1 variants that initially had no measurable thrombin-inhibitory potency into very strong thrombin inhibitors (Müller et al. 2020a). An exchange of the central globular domains of HLF2, HLF3l, and HLF4b with the central globular domain of HLF1 had the same effect (Müller et al. 2020b). Similar investigations on the HLFs of *Hirudinaria manillensis* are an excellent opportunity to figure out whether or not the observed structure–function relationships are unique to HLFs of the genus *Hirudo* or can be observed in HLFs of other genera as well and may hence represent a more general principle.

Some HLF genes may encode different mRNAs (and hence different HLF molecules) due to alternative splice events. For HLF3l/HLF3s such a mechanism could already be confirmed (Müller et al. 2016), and for HLF4a/HLF4b, it is likely (Müller et al. 2017, 2020b). In either case, the different splice events affect the junction between the third and the fourth exon. Consequently neither the N-termini nor the central globular domains of the respective factors are altered, but the lengths and the amino acid composition of their C-terminal tails are different. In case of HLF4, the C-terminal tail of HLF4b conveyed thrombin-inhibitory potency to the molecule, whereas the tail of HLF4a did not (Müller et al. 2020b). A similar different splice event with alternative products may be predicted for the HLF6 gene, too (GenBank Acc. No. KX215746). An mRNA/cDNA encoding HLF6 (now HLF6a) could already be identified, and the presence of a second splice variant (HLF6b) is still hypothetical. However, HLF6b would comprise a quite short but acidic C-terminal tail, the latter a characteristic structural feature of hirudins (Dodt et al. 1984; Scacheri et al. 1993). HLF6a, in contrast, has a very basic tail without any acidic amino acid residue.

So far, no evidence is available for the expression of an additional gene identified in *Hirudinaria manillensis*, the HLF7 gene (GenBank Acc. No. KX215747). According to the GT-AG rule of splicing, the gene comprises four exons and three introns, a typical feature of all hirudin and HLF genes. Again, different splice variants may be predicted at the junction between the third and the fourth exon, leading to the putative factors HLF7a and HLF7b. Both factors differ in length, amino acid composition, and *pI* value of their C-terminal tails.

Neither HLF6b nor HLF7a/b has been purified and functionally tested yet. Thus, the first aim of the present study was to close this gap. Furthermore, we wanted to elucidate the effects of alterations within the N-termini of HLF6b and HLF7a/b in combination with exchanges of the central globular domains on the thrombin-inhibitory potencies of the respective synthetic factors. Finally, we tried to determine the minimum length of the C-terminal tail of hirudins and HLFs that still retains the ability of the full protein to inhibit thrombin. The outcomes of these investigations may help to better understand the general principles of structure–function relationships in hirudins/HLFs and, in a broader perspective, to shed more light on the evolutionary background of the hirudin superfamily.

## Materials and methods

### Genotyping of animals and tissue preparation

The biological material used in this study (specimen of *Hirudinaria manillensis* and salivary gland preparations) was already described by Lukas et al. (2019). Information on genotyping data and GenBank accession numbers can be obtained from the same publication.

### Expression, factor Xa treatment, and purification of His‑tagged HLFs

The complete procedure to clone cDNAs encoding HLFs as well as to express, purify, and quantify the respective proteins was already previously described in great detail (Müller et al. 2016, 2020a, b). Briefly, we applied a system developed by Qiagen (Hilden, Germany). The pQE30Xa vector encodes a factor Xa protease recognition site between the His-tag coding region on the 5′ side and the multiple cloning site on the 3′ side. Factor Xa protease treatment allows to precisely cleave off the His-tag and results in a recombinant protein that is free of any unwanted vector-derived amino acids at the N-terminus.

Partial cDNAs of HLFs of interest were cloned into pQE30Xa and sequenced for accuracy, and clones of interest were transformed into appropriate *Escherichia coli* strains. After expression, cells were harvested and sonicated, and, following a centrifugation step, the supernatant was loaded onto a self-packed column containing Ni–IDA His-Bind® resin (Merck, Darmstadt, Germany). Washing and elution steps were performed as recommended by the manufacturer of the resin using buffers that contained increasing concentrations of imidazole. Equal volumes of every fraction were analyzed by SDS-PAGE on 20% gels for yield and purity of the respective HLF. Fractions of interest were dialyzed twice for 24 h at 4 °C against a 100-fold excess of factor Xa reaction buffer.

After dialysis, the HLFs were treated with factor Xa to cleave off the His-tag. Subsequently, factor Xa was removed using Xarrest™ agarose (EMD Millipore, Burlington, MA, USA). All steps were carried out as recommended by the manufacturer. Completeness of factor Xa and His-tag removal and purity of recombinant HLFs was confirmed by SDS-PAGE on 20% gels.

Where necessary, the concentration of a protein solution was increased using Vivacon 500 centrifugal units (Sartorius Stedim Lab Ltd., Stonehouse, UK) with a molecular weight cutoff (MWCO) of 2,000 g/mol (= kDa). Molar concentrations of final protein solutions were calculated by dividing the absorbance at 280 nm by the molar absorption coefficient according to the equation ε = (nW × 5,500) + (nY × 1,490) + (nC × 125) (Gill and von Hippel 1989; Pace et al. 1995).

### Blood coagulation assays

Thrombin time assays (TT; reference range 16.8–21.4 s) using a BFT II analyzer (Siemens Healthcare, Erlangen, Germany) were performed to verify the biological activity of purified HLFs. All steps were carried out as outlined by the manufacturer. The respective protein samples were diluted with dialysis buffer to reach final concentrations of 3.2 µmol/l or 0.32 µmol/l in the coagulation assays. Dade® Ci-Trol® 1 (Siemens Healthcare, Erlangen, Germany) was used as standardized human plasma. Measurements that exceeded 300 s were stopped and considered complete inhibition of clot formation. Statistical analyses were performed using the IBM SPSS v20.0 software package (IBM Deutschland GmbH, Ehningen, Germany).

### Generation of hybrid and synthetic HLF variants

The DNAs of the hybrid variants of HLF6 and HLF7 were generated using the gene synthesis service of Synbio Technologies (Monmouth Junction, NJ, USA). In addition, alterations in HLF variants were generated by PCR using appropriate primers. Table 1 summarizes the origin (either natural or synthetic) of all factors that are described and analyzed in the study.

**Table 1:**
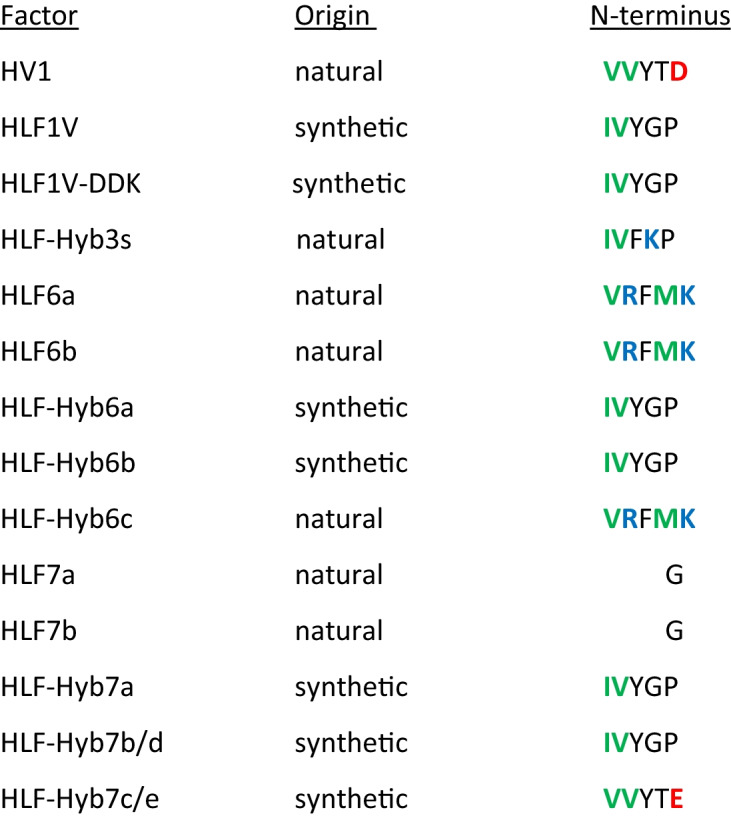
Origin (natural or synthetic) and N-terminal amino acid sequences of all factors described and analyzed in the study. Acidic residues are marked in red, basic residues are marked in blue, and neutral aliphatic residues are marked in green

## Results

The first aim of the present study was to functionally characterize both natural and synthetic variants of the hirudin-like factors HLF6 and HLF7 in their ability to inhibit thrombin.

### Functional analysis of HLF6 variants

**Fig. 1:**
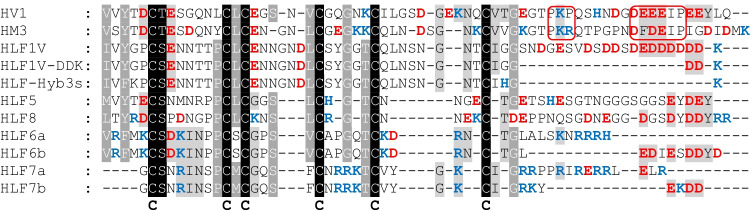
Multiple sequence alignment of hirudin variants HV1 of *Hirudo medicinalis* and HM3 of *Hirudinaria manillensis* and HLF variants HLF1V, HLF5, HLF8, HLF6, HLF6d, HLF7a, and HLF7b. The alignments were generated using the CLS Sequence Viewer software package v8.0 (CLC bio, Aarhus, Denmark). Black background indicates conserved residues; gray background indicates similar residues. The six conserved cysteine residues giving rise to the three dimensional structure are marked in bold, acidic amino acid residues are marked in red, and basic amino acid residues are marked in blue. The PKP and DFxxIP motifs are boxed. Abbreviations are used according to the IUPAC code

**Fig. 2:**
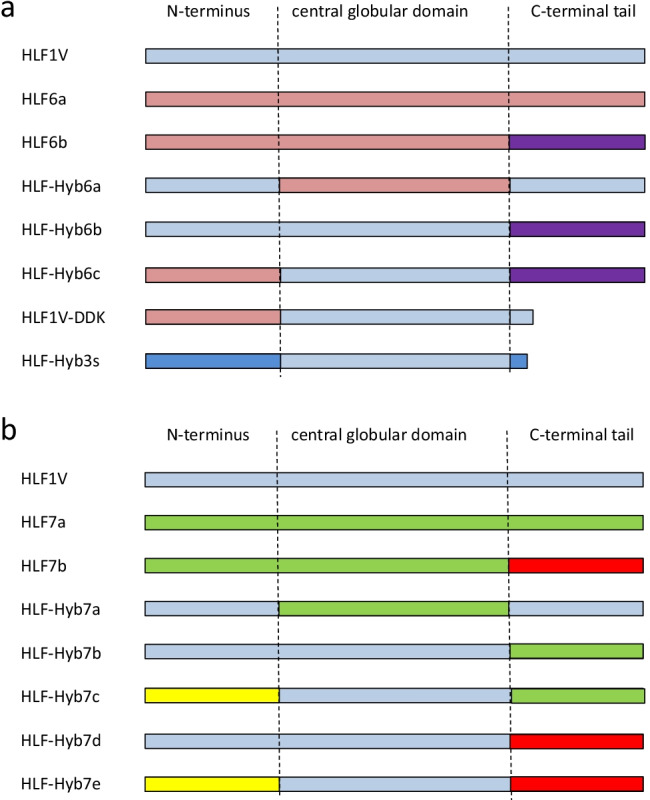
**a** Schematic representation of the N-termini, the central globular domains, and the C-terminal tails of HLF1V (light blue), HLF6a (light red), HLF6b (purple), HLF3s (dark blue), the hybrid variants HLF-Hyb6a-c, and the C-terminal truncated variants HLF1V-DDK and HLF-Hyb3s. **b** Schematic representation of the N-termini, the central globular domains, and the C-terminal tails of HLF1V (light blue), HLF7a (light green), and HLF7b (red) and the hybrid variants HLF-Hyb7a-e. The yellow color indicates an alternative N-terminus of HLF7-Hyb7c and HLF-Hyb7e derived from the nucleotide sequence of the HLF7 gene (see text for details)

**Fig. 3:**
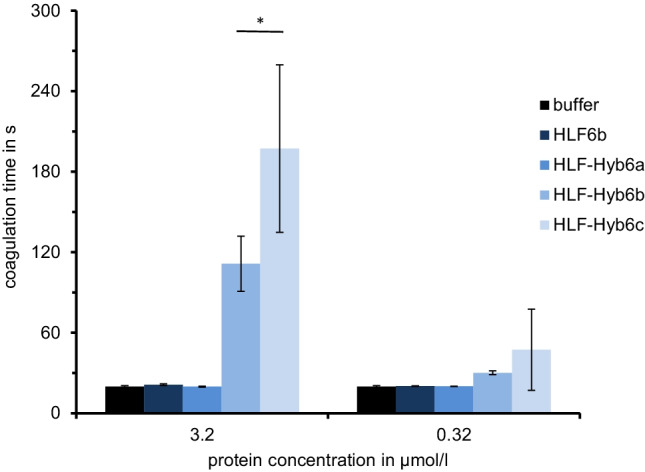
Standard blood coagulation assays using the thrombin time assay (TT) of HLF6d as well as the hybrids HLF-Hyb6a, HLF-Hyb6b, and HLF-Hyb6c. *n* = 3–5, error bars indicate SD. *: *p* < 0.05

The HLF6a and HLF6b cDNAs are putative splice variants of the same gene and encode proteins with identical N-termini and central globular domains, but different C-terminal tails (Fig. 1). HLF6a had already been functionally tested and did not inhibit thrombin (Lukas et al. 2019). According to previous investigations, the presence of “unsuitable” central globular domains or C-terminal tails render HLFs unable to inhibit thrombin (Müller et al. 2020a, b). We hence constructed three different hybrids of the highly active thrombin inhibitor HLF1V and HLF6b: HLF-Hyb6a (N- and C-termini of HLF1V fused to the central globular domain of HLF6b), HLF-Hyb6b (N-terminus and central globular domain of HLF1V fused to the C-terminal tail of HLF6b), and HLF-Hyb6c (N- and C-termini of HLF6b fused to the central globular domain of HLF1V) (Fig. 2a, Table 1 and Table 2). While HLF6b and HLF-Hyb6a displayed no thrombin-inhibitory potency in the thrombin time assay, HLF-Hyb6b and HLF-Hyb6c clearly did (Fig. 3). Hence, combination with the central globular domain of HLF1V converted the previously inactive HLF6b to a thrombin inhibitor. In that context, the inhibitory potency of HLF-Hyb6c (containing the N-terminus of HLF6a/b) was higher compared to HLF-Hyb6b (containing the N-terminus of HLF1V).

**Table 2:** Composition and thrombin-inhibitory potency of HLF6a/d and HLF7a/b and the HLF6 and HLF7 hybrids. The asterisk indicates a putative alternative N-terminus of HLF-Hyb7c/e derived from the nucleotide sequence of the HLF7 gene

Variant	N-terminus	Central domain	C-terminus	Inhibitory potency
HLF6a	HLF6	HLF6	HLF6a	No
HLF6b	HLF6	HLF6	HLF6b	No
HLF-Hyb6a	HLF1V	HLF6	HLF1	No
HLF-Hyb6b	HLF1V	HLF1	HLF6b	Medium
HLF-Hyb6c	HLF6	HLF1	HLF6b	Medium–high
HLF7a	HLF7	HLF7	HLF7a	No
HLF7b	HLF7	HLF7	HLF7b	No
HLF-Hyb7a	HLF1V	HLF7	HLF1	No
HLF-Hyb7b	HLF1V	HLF1	HLF7a	Low
HLF-Hyb7c	HLF7*	HLF1	HLF7a	Low-medium
HLF-Hyb7d	HLF1V	HLF1	HLF7b	Medium
HLF-Hyb7e	HLF7*	HLF1	HLF7b	Medium
HLF1V	HLF1V	HLF1	HLF1	Very high
HLF1V-DDK	HLF1V	HLF1	CIGG-DDK	Low
HLF-Hyb3s	HLF3	HLF1	HLF3s	Medium

### Molecular characterization of the HLF7 gene

The identification of the HLF7 gene was an unintended by-product in the course of our attempts to identify the gene of the hirudin-like factor HLF6 of *Hirudinaria manillensis*. An amplification product of about 650 bp in size was cloned and sequenced. Detailed sequence analyses revealed the presence of a gene encoding a yet unknown putative hirudin-like factor, named HLF7. The sequence of the HLF7 gene was deposited in GenBank under accession number KX215747.1. So far, no evidence for the expression of a respective HLF7 mRNA/cDNA in salivary gland cells of *Hirudinaria manillensis* could be obtained. However, the HLF7 gene comprises a structure that is typical for hirudin and HLF genes: four exons and three introns. The first exon is incomplete due to the localization of the forward primer within the coding region of the signal peptide sequence. The second and the third exons encode a central globular domain including the six conserved cysteine residues, whereas the fourth exon encodes a C-terminal tail. However, the exact splice position at the 5′end of exon 4 is uncertain. Two different alternative splice variants may be predicted when applying the classical GT-AG rule: HLF7a and HLF7b. Both variants differ from each other in terms of length and amino acid residue composition of the C-terminal tail. Whereas the tail of HLF7a is longer and basic (tail *pI* value of 12.12), the tail of HLF7b is shorter and slightly acidic (tail *pI* value of 6.12) (Fig. 1 and Table 2). The common central globular domain of both variants is basic (*pI* value of 9.18). The overall length (without the signal peptide) of HLF7a is 44 amino acid residues with a molecular mass of 5.2 kDa and a *pI* value of 10.76, whereas HLF7a has a length of 37 amino acid residues, a molecular mass of 4.2 kDa, and a *pI* value of 9.10. Most interestingly, the N-termini of HLF7a/b comprise only a single amino acid, a glycine residue (Fig. 1). With only five nucleotide exchanges, however, a putative alternative N-terminus of HLF7a/b (VVYTE) can be deduced from the boundary of intron 1 and exon 2 that strongly resembles the N-terminus of hirudin HV1 (VVYTD).

### Functional analysis of HLF7 variants

**Fig. 4:**
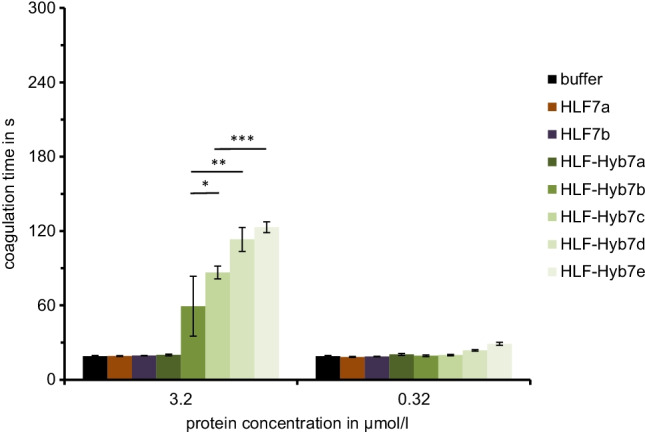
Standard blood coagulation assays using the thrombin time assay (TT) of HLF variants HLF7a and HLF7b as well as the hybrids HLF-Hyb7a, HLF-Hyb7b, and HLF-Hyb7c. *n* = 3–5, error bars indicate SD. *: *p* < 0.05; **: *p* < 0.01; ***: *p* < 0.001

For the functional analysis of putative HLF7 variants, we constructed respective cDNAs of HLF7a and HLF7b by gene synthesis. In addition, we constructed the following hybrid variants of either HLF7a or HLF7b and HLF1V: HLF-Hyb7a (N- and C-termini of HLF1V fused to the central globular domain of HLF7a/b), HLF-Hyb7b (N-terminus and central globular domain of HLF1V fused to the C-terminal tail of HLF7a), HLF-Hyb7c (alternative N-terminus of HLF7a/b fused to the central globular domain of HLF1V and the C-terminal tail of HLF7a), HLF-Hyb7d (N-terminus and central globular domain of HLF1V fused to the C-terminal tail of HLF7b), and HLF-Hyb7e (alternative N-terminus of HLF7a/b fused to the central globular domain of HLF1V and the C-terminal tail of HLF7b) (Fig. 2b, Table 1 and Table 2). Neither HLF7a nor HLF7b had any inhibitory effect on thrombin in the thrombin time assay, and the same was observed for HLF-Hyb7a (Fig. 4). In contrast, all the other hybrid variants of HLF7a/b were thrombin inhibitors. In detail, the more acidic C-terminal tail of HLF7b conferred a higher thrombin-inhibitory potency compared to the more basic C-terminal tail of HLF7a (compare HLF-Hyb7d/e to HLF-Hyb7b/c, Fig. 4), and the alternative N-terminus of HLF7a/b (VVYTE) was at least as effective in terms of thrombin inhibition compared to the N-terminus of HLF1V (IVYGP) (compare HLF-Hyb7c/e to HLF-Hyb7b/d, Fig. 4).

### Minimum length of the C-terminal tail

**Fig. 5:**
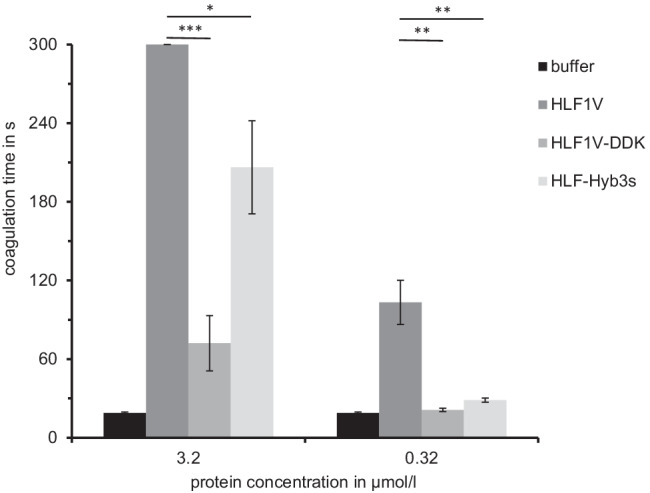
Standard blood coagulation assays using the thrombin time assay (TT) of HLF1V, HLF1V-DDK, and HLF-Hyb3s. *n* = 3–5, error bars indicate SD. *: *p* < 0.05; **: *p* < 0.01; ***: *p* < 0.001

The C-terminal tails of HLF6b and HLF7a/b are considerably shorter compared to the tails of hirudins (e.g., HV1 of *Hirudo medicinalis* or HM3 of *Hirudinaria manillensis*) or HLF1V, HLF5, and HLF8 (Fig. 1 and Table 3) but still enabled thrombin inhibition (see Figs. 3 and 4). The second aim of the present study was hence to estimate the minimum length of the C-terminal tail of hirudins/HLFs that still confers the ability of the full protein to inhibit thrombin. Two strategies were applied. First, we constructed the following “short tail variants” of the highly effective thrombin inhibitor HLF1V (C represents the sixth cysteine residue, Fig. 1): CIGG-DDK/-DK/-DD/-D/-K/-, respectively. Of these constructs, only the variant “CIGG-DDK” (Figs. 1 and 5a) could be successfully expressed, purified, and processed in an amount that was sufficiently high for the subsequent functional analysis. Based on that observation, we applied a second strategy and constructed a hybrid of HLF3s and HLF1 (HLF-Hyb3s, N- and C-termini of HLF3s combined with the central globular domain of HLF1, Table1, Fig. 1, and Fig. 2a). In a previous study, we were able to successfully express and purify HLF3s, but the factor did not inhibit hirudin (Müller et al. 2020b). As shown in Fig. 5, both HLF1V-DDK and HLF-Hyb3s clearly are thrombin inhibitors, even with the short C-terminal tails of only six (HLF1V-DDK) or four (HLF-Hyb3s) amino acid residues in length. Their thrombin-inhibitory potencies, however, are lower compared to HLF1V.

**Table 3:** Molecular properties of the C-terminal tails of hirudin and HLF variants. Number indicates the number of amino acid residues starting immediately after C6 (the sixth cysteine residue). The acidic/basic ratio indicates the number of acidic and basic amino acid residues together with the deduced *pI* value

Factor	Number	Acidic/basic	*pI* value
HV1	26	8/2	3.94
HM3	28	5/4	4.78
HLF1V	24	13/1	3.26
HLF1V-DDK	6	3/1	4.21
HLF-Hyb3s	4	0/2	8.76
HLF5	23	5/1	3.90
HLF8	24	8/2	3.76
HLF6a	12	0/5	12.30
HLF6b	12	6/0	3.25
HLF7a	16	2/7	12.12
HLF7b	9	3/3	6.12

## Discussion

In recent investigations, we have analyzed and functionally characterized HLFs that are present in European medicinal leeches, namely HLF1, HLF2, HLF-Hyb, HLF3, and HLF4 (Müller et al. 2016, 2020a, b). Modifications within the N-termini of the respective factors have the potential to dramatically alter (increase or decrease) their thrombin-inhibitory potencies. These results are in agreement with examinations of the N-termini of hirudins like HV1 of *Hirudo medicinalis* (Betz et al. 1992; Wallace et al. 1989; Lazar et al. 1991) or HM1 of *Hirudinaria manillensis* (De Filippis et al. 1995, 1998). In addition, the construction and functional characterization of hybrid variants of HLF1 on one hand and HLF2, HLF3, or HLF4 on the other hand revealed strong evidence for the crucial importance of the central globular domain of HLFs on the thrombin-inhibitory potency of the whole molecule as well (Müller et al. 2020a, b). In the current work, we have applied a similar strategy and technical approach to the functional characterization of the hirudin-like factors HLF6b and HLF7a/b of the Asian medicinal leech, *Hirudinaria manillensis*.

Both factors may occur in two different splice variants, namely HLF6a/HLF6b or HLF7a/HLF7b, respectively. None of these factors displayed a significant thrombin-inhibitory potency when analyzed in the thrombin time assay. The same was observed with hybrid factors that contain the central globular domain of either HLF6 or HLF7. Thrombin inhibition was only achieved with constructs containing the central globular domain of HLF1 instead of those of HLF6 or HLF7, giving rise to the hybrid factors HLF-Hyb6b/c and HLF-Hyb7b-e, respectively (Fig. 3 and Fig. 4). These observations support our hypothesis that all parts of the hirudin/HLF molecules are of crucial importance for their ability to inhibit thrombin, a general principle that obviously holds true not only for HLFs of the European medicinal leeches, but also for the respective factors of the Asian medicinal leech, *Hirudinaria manillensis*.

However, the exact composition of the N- and C-termini is important as well. Compared to HLF1V, the N-terminus of HLF6 seems to work better: the hybrid variant HLF-Hyb6b comprising the N-terminus of HLF1V (IVYGP) has a lower inhibitory potency compared to the otherwise identical hybrid variant HLF-Hyb6c comprising the N-terminus of HLF6 (VRFMK). A similar observation was made for the N-termini of HLF2 and HLF3 (Müller et al. 2020a, b).

On the other hand, the C-terminal tail of HLF1 confers a higher thrombin-inhibitory potency compared to the C-termini of both HLF6b and HLF7a/b (Figs. 3, 4, and 5). These differences are likely the result of differences in both length and charge of the respective tails. The C-terminal tail of hirudin interacts with the exosite 1 of thrombin and hence blocks the binding of the substrate fibrinogen (Maraganore et al. 1989; Fenton et al. 1991; Rydel et al. 1991). Several ionic and non-ionic interactions between hirudin and thrombin (Braun et al. 1988; Betz et al. 1991b; Huang et al. 2014) including the presence of numerous acidic amino acid residues (7 out of 29 residues in HV1, 9 out of 27 residues in HM1) (Betz et al. 1991a) are of particular importance in that context. The C-terminal tail of HLF1V contains even more acidic amino acid residues (13 out of 24 compared to HV1), whereas the tails of HLF5 and HLF8 are slightly less acidic (Fig. 1). As an overall impression, the C-terminal tails of hirudins/HLFs are obviously much less constrained in their molecular characteristics: a charged character including the presence of several acidic amino acids seems to be sufficient for thrombin inhibition (Müller et al. 2020a, b). The C-terminal tails of HLF6b and HLF7a/b support this observation. Both are shorter compared to HV1 and HLF1V but contain several charged amino acid residues (Fig. 1, Table 3). However, the tails of HLF6b (12 amino acid residues and a *pI* value of 3.25) and HLF7b (9 amino acid residues and a *pI* value of 6.12) are shorter, but more acidic compared to the tail of HLF7a (16 amino acid residues and a *pI* value of 12.12). Consequently, molecules containing the tails of HLF6b and HLF7b exhibited higher thrombin-inhibitory potencies compared to those containing the tail of HLF7a (Fig. 3 and Fig. 4). However, even the basic tail of HLF7a does not entirely alleviate thrombin-inhibitory potency.

The minimum length of a C-terminal tail of hirudins/HLFs that still confers thrombin-inhibitory potency to the full molecule could not yet exactly be determined, given that both HLF1V-DDK and HLF-Hyb3s are still thrombin inhibitors (Fig. 5). HLF1V variants with shorter tails could not be successfully expressed in *Escherichia coli* cells (several strains were tested), a phenomenon that was probably due to C-terminal protein instability: the composition of the C-terminus strongly influences protein stability in bacterial cells (Parsell et al. 1990; Weber et al. 2020). C-terminally truncated variants of hirudin HV1 occur in vivo as a result of metabolic degradation in the kidney (the so-called urohirudin; Nowak and Schrör 2007) or have been generated in vitro by exopeptidase-mediated proteolysis (Chang 1983; Dodt et al. 1987). As a consequence, the thrombin-inhibitory potency of truncated hirudin HV1 is either drastically reduced (deletions of 3–7 amino acid residues) or almost completely lost (deletions of 16–22 amino acid residues). The data for HLF1V-DDK are in good agreement with these observations.

The differences in the thrombin-inhibitory potencies of HLF1V and HLF1V-DDK are of great potential for a rational molecule design. It seems plausible that the length of the C-terminal tail of HLF1V, in particular the number of acidic amino acid residues, will directly correlate with the level of thrombin-inhibitory potency of the respective variants. A stepwise truncation may result in a set of factors whose inhibitory potencies on thrombin and hence therapeutic effects may easily be adjusted to the specific needs and conditions of an individual patient in the hospital. To verify this may be a challenging, but promising task for the future.

The comparably high thrombin-inhibitory potency of HLF-Hyb3s was a somehow surprising observation. HLF-Hyb3s was as effective in thrombin inhibition as its direct counterpart HLF-Hyb3b despite the much shorter C-terminal tail (Müller et al. 2020b). This raises the question whether HLF-Hyb3s (and HLF-Hyb3b as well) is still a bi- or rather a mono- (or uni-) valent inhibitor of thrombin. In the latter case, both factors would interact with the reactive center of thrombin only, but not with the exosite 1. Detailed structural analyses based on co-crystallization experiments with thrombin could clarify this highly interesting point.

## Data Availability

All relevant sequence data were deposited in GenBank for public access.
